# Supplementary material for: Association between oral microbiome and seven types of cancers in East Asian population: a two-sample Mendelian randomization analysis
Source: Front Mol Biosci. 2023 Nov 21;10:1327893. doi: 10.3389/fmolb.2023.1327893 (PMC10702768; doi:10.3389/fmolb.2023.1327893)
Supplement: Supplementary file 1 [file DataSheet1.ZIP › Supplementary Materials/MR plots of saliva/saliva═╝/ovarian cancer/pheno.2172_to_ovarian cancer_leave_one_out.pdf]

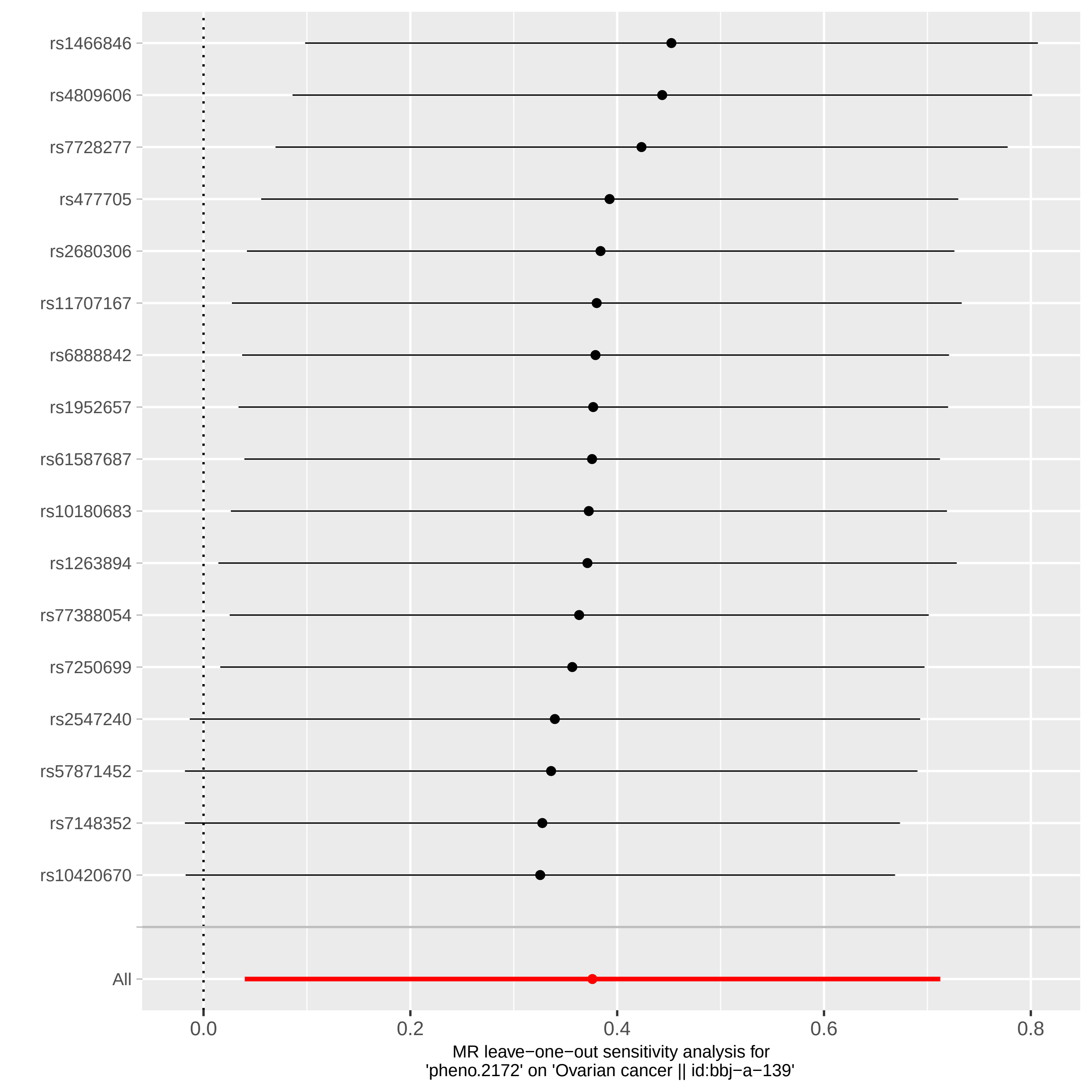
rs1466846
rs4809606
rs7728277
rs477705
rs2680306
rs11707167
rs6888842
rs1952657
rs61587687
rs10180683
rs1263894
rs77388054
rs7250699
rs2547240
rs57871452
rs7148352
rs10420670
All
0.0
0.2
0.4
0.6
0.8
MR leave−one−out sensitivity analysis for
'pheno.2172' on 'Ovarian cancer || id:bbj−a−139'
